# Prolonged Physiotherapy after Anterior Cruciate Ligament Reconstruction Does Not Improve Muscular Strength and Function

**DOI:** 10.3390/jcm13092519

**Published:** 2024-04-25

**Authors:** Marc Dauty, Emmanuel Le Mercier, Pierre Menu, Jérôme Grondin, Thomas Hirardot, Pauline Daley, Alban Fouasson-Chailloux

**Affiliations:** 1 Physical Medicine and Rehabilitation, Sport Medicine Center, University Hospital of Nantes, CHU Nantes, 44093 Nantes, France; marc.dauty@chu-nantes.fr (M.D.); emmanuel.lemercier@chu-nantes.fr (E.L.M.); pierre.menu@chu-nantes.fr (P.M.); thomas.hirardot@chu-nantes.fr (T.H.); 2 Institut Régional de Médecine du Sport des Pays de Loire (IRMS), 44093 Nantes, France; paulinedaleyy@gmail.com; 3 Inserm, UMR 1229, RMeS, Regenerative Medicine and Skeleton, ONIRIS, Nantes Université, 44042 Nantes, France

**Keywords:** anterior cruciate ligament, surgery, sport, knee, injury, rehabilitation

## Abstract

**Background:** After the rupture of the anterior cruciate ligament (ACL), surgery is proposed in the case of knee instability or for athletes who want to return to a pivotal and/or contact sport. The current trend is to extend physiotherapy sessions until a patient’s return to sport. We aimed to assess the interest in prolonging the physiotherapy sessions up to 4 postoperative months to restore muscle knee strength and function. **Methods:** From a historical cohort, 470 patients (24.3 ± 8.7 years) were included; 312 (66%) were males. They all had undergone a primary ACL reconstruction with a hamstring procedure. The number of physiotherapy sessions was established at 4 postoperative months. The main study parameters to assess the benefit of prolonged physiotherapy were the isokinetic limb symmetry index (LSI) for the quadriceps and the hamstrings as well as the Lysholm score. **Results:** At 4 postoperative months, 148 patients (31.4%) still had physiotherapy sessions. This group had performed 49 ± 14 physiotherapy sessions at the time of evaluation compared to 33 ± 9 sessions performed by the group that stopped physiotherapy at 3 months post-ACL reconstruction. The isokinetic knee LSI and the Lysholm score were not different between the two groups. Continued physiotherapy sessions were associated with female gender, previous high sport level, meniscal repair, lateral tenodesis and outpatient rehabilitation at the beginning of the rehabilitation management, while knee pain complications were not associated. **Conclusions:** No significant correlation was found between the number of physiotherapy sessions and the knee strength LSI or the Lysholm score. Prolonging patient physiotherapy sessions after 3 months post-ACL reconstruction seems ineffective in improving knee strength recovery and function.

## 1. Introduction

After the rupture of the anterior cruciate ligament (ACL), surgery is proposed in case of knee instability or for athletes who want to return to a pivotal and/or contact sport [1]. Currently, the hamstring procedure is often performed, even if other grafts can also be used [2]. Then, a long rehabilitation process is necessary to recover a painless, full range of motion and a stable knee, to return to professional and sporting activities [3]. Immediately after ACL reconstruction (ACLR), knee swelling is responsible for arthrogenic muscle inhibition (AMI), which is clinically expressed through muscle strength inhibition, particularly on the knee extensors (quadriceps) [4,5]. Early rehabilitation aims at decreasing knee swelling to reduce pain, to help recover full range of motion and to restore muscle strength [6]. The absence of complications at the knee level and delays in the ACLR seem to play a significant role in the recovery process [7], as does the number of rehabilitation sessions [8]. Yet, some patients only have a few sessions of physiotherapy, whereas some others have more than 60, with no obvious benefits concerning knee joint recovery [8,9]. However, the current trend is to extend physiotherapy sessions until a patient’s return to sport, as it is carried out for professional athletes. Yet, at 3 postoperative months, the patient’s return to running is often authorized by surgeons according to temporal criteria [10] and criteria linked to the recovery of the joint, which must be painless and mobile, with no effusion. At this time, the muscular strength of the operated knee is still deficient, as evidenced by the isokinetic limb symmetry index (LSI) of the extensors, at around 70%, which is a mark of the persistence of joint muscular inhibition. It has been established that at 4 months after surgery, the threshold of 65% for the LSI of the quadriceps could help authorize a patient’s return to running [11]. Continuing rehabilitation sessions could, therefore, be justified in improving joint muscle inhibition. However, the number of these physiotherapy sessions remains debated [12]. Indeed, it has been retrospectively shown that the duration of the physiotherapy period was correlated with the recovery of the LSI of the knee extensors when this period was greater than 6 months after surgery or included 60 sessions [8,13]. Yet, conversely, recent meta-analyses have shown that frequent supervised physiotherapy sessions do not provide better results in improving knee muscle strength [14,15].

We hypothesized that physiotherapy sessions up to 4 months after surgery could be more effective than physiotherapy stopped after 3 months in recovering from muscular inhibition of the knee. So, in this work, we aimed to assess the interest in prolonging the physiotherapy sessions up to 4 postoperative months to restore muscle knee strength and function.

## 2. Materials and Method

### 2.1. Study Design

We performed a retrospective analysis based on a prospective cohort of patients who underwent ACLR between 2020 and 2022. We assessed all the patients referred to the sports medicine department of a university hospital for their inclusion eligibility to participate in an isokinetic evaluation after ACLR.

### 2.2. Patients

From a historical cohort limited to 2 years [2020–2022], 470 patients ≥18 years old (24.3 ± 8.7 years [18–50 years]) were included. They all had undergone a primary ACLR with a hamstring procedure (Semitendinosus or Semitendinosus-Gracilis). The surgeries were performed by several different experienced surgeons, working either in public hospitals or private clinics. The exclusion criteria were knee complications such as arthrofibrosis or cyclops syndrome (complications limiting the knee range of motion in the long term), thrombophlebitis or postoperative infection [16,17]. Gender, age, weight, height, sport, sport level before surgery and concomitant surgical procedures (meniscal repair, lateral tenodesis) were considered. The sport level before ACLR was assessed using the Tegner activity scale according to a dichotomic evaluation: ≥9 or <9 [18]. A score ≥9 was considered in the case of a patient’s previous practice of sports like soccer, basketball, handball, gymnastics, motocross and fighting at an elite sport level (national, international or professional). Postoperative complications such as anterior and posterior knee pain have been collected [7,19]. The method was conducted in respect of the Helsinki declaration for retrospective study after the approval by the local ethical comity, Comité Nantais d’Ethique en Médecine du Sport of the Regional Institute of Sports Medicine, on 7 November 2023 (register number: CNEMS-2023-001).

### 2.3. Physiotherapy Sessions

The number of physiotherapy sessions was established at 4 postoperative months by interviewing the patients. The nature of the sessions and the techniques used have not been assessed, even if all the patients had an initial prescription for an accelerated rehabilitation program after surgery, avoiding knee joint immobilization [20]. If the physiotherapy began during inpatient rehabilitation, 10 sessions were considered to have been performed per week. The cohort was then divided into 2 groups, the first had stopped their physiotherapy sessions before 3 completed postoperative months, and the second had continued their physiotherapy until the isokinetic assessment of the knee muscle strength at 4 postoperative months. The cessation of the sessions was linked to the initial prescription of the surgeon and independent of the evaluator. Yet, our 3- and 4-month time-point assessments were chosen according to the literature findings. On one hand, at 3 months, the patient’s return to running is often authorized by surgeons according to temporal criteria [10], and on the other hand, at 4 months, the isokinetic threshold of 65% for the quadriceps’ LSI could help to authorize the patient’s return to running [11].

### 2.4. Main Outcomes

The main study parameters to judge the benefit of prolonged physiotherapy were the isokinetic limb symmetry index (LSI) for the quadriceps and the hamstrings [21] and the Lysholm score [18]. Isokinetic measurements were performed in a sitting position to assess the knee joints using a Humac^®^ isokinetic dynamometer (Medimex, Sainte-Foy-lès-Lyon, France). Every session was preceded with a familiarization with the isokinetic movement (3 submaximal movements). The patients were tested over 3 maximal repetitions at the angular speed of 60°/s followed by 5 maximal repetitions at 180°/s, according to our clinical habits and as previously published [11,21]. A 30 s recovery period was allowed between both series. The uninvolved limb was always evaluated first, after instruction, and with verbal encouragement. All the evaluations were conducted by the same sports physician away from the surgeons. The quadriceps and hamstring limb symmetry indices (Q-LSI and H-LSI) were expressed in percentages and calculated with the following formula: (Peak torque of the surgical limb/peak torque of the uninvolved limb) × 100. The reliability of the quadriceps LSI and the hamstring LSI was considered acceptable (ICC: 0.43–0.78) [22]. The Lysholm score measures the functional impact in terms of disabilities and activity limitations. Eight items are reported for a maximal score of 100 points. A global score of less than 65 points was considered low, while a score of more than 84 was a high score. The Lysholm score has been described as reliable and valid for functional assessment after ACLR [23].

### 2.5. Statistical Analysis

The number of subjects required for the study was calculated (Biostatgv^®^ project, Paris, France) to obtain a clinical difference of 10% between the 2 groups (continuous and non-continuous physiotherapy) for the LSI of the knee extensors set at 65% (angular velocity of 60°/s with a first-species risk α = 5% and a power of 1 − β = 90%). The value of 65% was considered with reference to the cut-off established by Grondin et al., allowing the patient to return to running [11]. The total number of patients included was 48 per group. The two groups were compared using the SPSS 23.0^®^ software package (Armonk, NY, USA). The quantitative variables comparison was performed using a *t*-test after the verification of the equality of the variances according to the Levenne test. The qualitative variables were compared using the χ^2^ test. To identify independent predictors of prolonged physiotherapy sessions, a multivariate analysis was performed using ascendant Wald logistic binary regression (prolonged physiotherapy or not). Odds ratios [Exp(Ɓ)] and 95% CIs were estimated [24]. To determine links between the number of physiotherapy sessions and the isokinetic LSI or the functional score, Pearson’s product-moment correlation analysis was performed. The alpha level of statistical significance was set at 0.05.

## 3. Results

At 4 postoperative months, 148 patients (31.4%) still had physiotherapy sessions. This group had performed 49 ± 14 physiotherapy sessions at the time of evaluation compared to 33 ± 9 sessions performed by the group that stopped physiotherapy at 3 months post-ACLR (Table 1). The isokinetic knee LSI and the Lysholm score were not different between the two groups at 4 post-ACLR months (Table 2).

Continued physiotherapy sessions were associated with female gender, previous high sport level (Tegner activity scale ≥ 9), meniscal repair, lateral tenodesis and outpatient rehabilitation at the beginning of the rehabilitation management, while knee pain complications were not associated (Table 3). No significant correlation was found between the number of physiotherapy sessions and the knee strength LSI or the Lysholm score.

## 4. Discussion

Our study aimed to determine whether physiotherapy sessions carried out for up to 4 postoperative months were more effective than physiotherapy stopped after 3 postoperative months to recover from the muscular inhibition of the operated knee, calculated using the LSI. The difference in duration of postoperative physiotherapy, 4 months instead of 3 months, was logically responsible for carrying out a greater number of sessions (*p* = 0.001). However, despite these findings, muscle inhibition was not different at 4 postoperative months between the two groups. So, according to our results, there seems to be no interest in prolonging the physiotherapy after 3 months post-ACLR to improve the AMI assessed using the isokinetic strength LSI and the Lysholm functional score. Indeed, the AMI corresponds to a knee protection reflex mechanism that decreases over time [5]. This is a well-known phenomenon following knee surgery or injury [4]. This is a reflex inhibition of the muscles surrounding the knee, after distension or damage to the joint, which is due to articular swelling, inflammation, and pain. The strength deficit may still persist for several months after ACLR. So, it is the muscle strength recovery thanks to the AMI decrease that allows the muscle strengthening exercises or the return to physical activities, and not the reverse. This probably explains the lack of correlation found between the number of physiotherapy sessions and the knee strength LSI, as recently reported by Czamara et al. [8]. However, our results are difficult to compare with those of the literature because of the small number of studies carried out on the subject, with a predominance of works by the same medical team [8,25,26]. Indeed, the number of sessions, from 4 to more than 60, is very variable and involves the time effect when the subjects have only completed 10 rehabilitation weeks against more than 28 weeks [25]. In addition, the group that performed fewer sessions (<5) is often referred to as a non-compliant group. So, it is not surprising that this group has poorer results in terms of function (Lysholm score) and returning to sport compared to a compliant group that completed more than 15 sessions [9]. The content of the sessions is also questionable, particularly with the use of the term “muscle strengthening” without knowing if it is rather a fight against muscular inhibition. Otherwise, we tried to explain what could have led to continuing the physiotherapy sessions up to 4 post-ACLR months by looking for associations with some parameters that we had collected. The continuous prescription of physiotherapy sessions was associated with concomitant surgery (meniscal repair and lateral tenodesis), female sex, pre-existing high-level sport and outpatient management. Several hypotheses, such as fears and beliefs, can be elaborated to explain these associations [27]: (1) on the part of the surgeons, when a surgical procedure was associated or when hyperlaxity was present, particularly in women; (2) on the part of the patients, who had previously been high-level athletes and who want permanent medical supervision, possibly for fear of a new knee injury and (3) on the part of the physiotherapists (outpatient care) who may have an interest in offering care until the patient’s return to sport. On the other hand, it is surprising that continuous physiotherapy care was not associated with postoperative knee pain, whereas these complications are debilitating. Concerning the association with lateral tenodesis, the explanation is complex given that this surgical procedure is not consensual and is often associated with the preoperative presence of a pivot shift and the high-level status of the patient, practicing a high-risk sport for the knee such as pivot sports with contact [28,29,30]. Unfortunately, the explanations offered are only hypotheses since we do not know what and/or who exactly influenced the prescription prolongation of the physiotherapy sessions. In the future, patient-specific adjustments may be needed to improve compliance while managing knee pain. Psychological, and maybe financial, changes could, therefore, be considered because of the lack of objective evidence to extend the physiotherapy duration.

### Limitations

The rationale for the initial physiotherapy prescription duration was not known. It would have been interesting to study what influenced the continuation of physiotherapy sessions up to 4 postoperative months according to the associated surgical procedures. The content and the quality of the physiotherapy sessions were not reported even if all patients had a prescription for accelerated rehabilitation [21]. These sessions were undoubtedly adapted according to the operated knee and the patient. Thus, it is difficult to know if each of the sessions was truly optimal for reducing one’s muscular inhibition of the knee. However, muscle strength related to the arthrogenic inhibition of the quadriceps and the hamstrings is closely linked to the time parameter [4]. Furthermore, we did not study the psychosocial parameters, which may have influenced the performance, frequency and prolongation of physiotherapy sessions [12,27,31]. Our results are also probably not comparable to those of other countries, due to different healthcare systems. In fact, in Canada and the United States, the number of physiotherapy sessions is lower, with a median of 16 (from 9 to 22), and 90% of them are performed during the first 4 postoperative months [32]. This number is lower than for our group that had the fewest sessions.

## 5. Conclusions

Prolonging the physiotherapy sessions after 3 months post-ACL reconstruction seems ineffective in improving knee strength recovery and function. Only about thirty sessions could be necessary in the absence of severe complications after ACL reconstruction. In the future, studies should focus on the nature and quality of the physiotherapy sessions to assess the impact of the techniques used.

## Figures and Tables

**Table 1:** Comparison of continued and non-continued physiotherapy groups at baseline.

	Continued Physiotherapy Group (n = 148)	Non-Continued Physiotherapy Group (n = 322)	*p*
Gender			0.006 *
Male, n (%)	85 (57.4%)	227 (70.5%)	
Female, n (%)	63 (42.6%)	95 (29.5%)	
Age, year	23.4 ± 8.8	24.8 ± 8.7	0.09
Weight, kg	71.4 ± 17.0	71.9 ± 16.0	0.79
Height, cm	171.0 ± 9.0	173.0 ± 8.0	0.28
ACL injury-ACLR duration, days	189.0 ± 185	207.0 ± 325.0	0.53
Sport level before ACLR			0.0001 *
Tegner activity score ≥ 9, n (%)	35 (23.7%)	34 (10.6%)	
Tegner activity score < 8, n (%)	113 (76.3%)	288 (89.4%)	
Concomitant surgery:			0.001 *
Meniscus repair			
Yes, n (%)	56 (37.8%)	73 (22.7%)	
No, n (%)	92 (62.2%)	249 (77.3%)	
Lateral tenodesis			0.001 *
Yes, n (%)	56 (37.8%)	40 (12.4%)	
No, n (%)	92 (62.2%)	282 (87.6%)	
Rehabilitation procedure			0.001 *
Inpatient, n (%)	67 (45.2%)	206 (63.9%)	
Outpatient, n (%)	81 (54.8%)	116 (46.1%)	
Complications			0.43 *
Yes, n (%)	34 (22.9%)	78 (24.2%)	
No, n (%)	114 (77.1%)	244 (75.8%)	
Physiotherapy sessions, n	49 ± 14	33 ± 9	0.001

Abbreviations: *: χ^2^ test; ACLR: Anterior Cruciate Ligament Reconstruction.

**Table 2:** Comparison of isokinetic knee strength LSI and Lysholm score at 4 post-ACLR months between continued and non-continued physiotherapy groups.

	Continued Physiotherapy Group (n = 148)	Non-Continued Physiotherapy Group (n = 322)	*p*
ACLR-Isokinetic testing, days	120 ± 16	120 ± 15	0.87
Q60-LSI, %	70 ± 16	72 ± 16	0.16
H60-LSI, %	81 ± 14	82 ± 13	0.45
Q180-LSI, %	76 ± 14	78 ± 15	0.17
H180-LSI, %	81 ± 15	84 ± 17	0.06
Lysholm score	94 ± 8	94 ± 8	0.74

Abbreviations: Q60-LSI: Quadriceps Limb Symmetry Index at 60°/s; H180-LSI: Hamstring Limb Symmetry Index at 180°/s; ACLR: Anterior Cruciate Ligament Reconstruction.

**Table 3:** Continued physiotherapy rehabilitation model to identify associated factors (binary logistic regression).

Factors	Béta	Wald	OR	95% CI	*p*
Lateral tenodesis	1.24	23	3.45	2.09–5.68	0.001
Previous sport level ≥ 9	1.04	12	2.85	1.58–5.15	0.001
Outpatient	0.85	13	2.34	1.48–3.69	0.001
Meniscal repair	0.65	7.6	1.91	1.20–3.04	0.006
Female gender	0.57	6.4	1.77	1.14–2.75	0.01
Constant	−2.3	31			

Abbreviations: OR: odds ratio; CI: confidence interval.

## Data Availability

The data presented in this study are available upon request from the corresponding author. The data are not publicly available due to ethical reasons.
